# New Tricks for an Old (Hedge)Hog: Sonic Hedgehog Regulation of Astrocyte Function

**DOI:** 10.3390/cells10061353

**Published:** 2021-05-30

**Authors:** A. Denise R. Garcia

**Affiliations:** Department of Biology, Drexel University, Philadelphia, PA 19104, USA; adg82@drexel.edu

**Keywords:** Sonic hedgehog, astrocyte, glia, inflammation, neuron–astrocyte communication

## Abstract

The Sonic hedgehog (Shh) molecular signaling pathway is well established as a key regulator of neurodevelopment. It regulates diverse cellular behaviors, and its functions vary with respect to cell type, region, and developmental stage, reflecting the incredible pleiotropy of this molecular signaling pathway. Although it is best understood for its roles in development, Shh signaling persists into adulthood and is emerging as an important regulator of astrocyte function. Astrocytes play central roles in a broad array of nervous system functions, including synapse formation and function as well as coordination and orchestration of CNS inflammatory responses in pathological states. Neurons are the source of Shh in the adult, suggesting that Shh signaling mediates neuron–astrocyte communication, a novel role for this multifaceted pathway. Multiple roles for Shh signaling in astrocytes are increasingly being identified, including regulation of astrocyte identity, modulation of synaptic organization, and limitation of inflammation. This review discusses these novel roles for Shh signaling in regulating diverse astrocyte functions in the healthy brain and in pathology.

## 1. Introduction

Hedgehog (Hh) signaling has long been recognized as a key molecular signaling pathway orchestrating nervous system development. Initially identified in *Drosophila* for its role in segmentation and patterning of developing larvae [1], the pathway is evolutionarily conserved, and its vertebrate homolog, Sonic hedgehog (Shh), exerts powerful regulation over various aspects of vertebrate neurodevelopment. Shh signaling establishes the ventral identity of cells in the neural tube of early embryos [2] and is required for specifying ventral neuron and oligodendrocyte cell fate of uncommitted neural progenitors [3,4,5,6]. Shh also functions as an axon guidance molecule, directing developing axons towards appropriate target areas [7,8,9,10]. In the cerebellum, Shh acts as a mitogen, regulating proliferation of granule cell precursors [11,12]. However, Shh signaling persists into adulthood throughout the brain, where it is transduced in astrocytes [13,14], one of two primary classes of macroglial cells in the central nervous system (CNS). Whereas Shh signaling during development regulates the behaviors of neural precursor and progenitor cells as well as immature neurons, astrocytes are fully differentiated, postmitotic cells, suggesting that its functional role in these cells is likely to differ substantially from that in development. Indeed, emerging evidence shows that Shh signaling plays a role in astrocyte modulation of synaptic function in the healthy brain and also restricts inflammation under pathological conditions. In addition to astrocytes, Shh signaling is also found in adult neural stem cells residing in the subventricular zone (SVZ) and hippocampus, where its activity is required to maintain their self-renewal [15,16,17]. In this review, we focus on the emerging work uncovering the role of Shh signaling in astrocytes.

Early studies identified multiple components of the Shh signaling pathway throughout several regions of the CNS [18,19]. Much of the early focus on Shh signaling in the adult CNS centered on its role in regulating adult neural stem cell (NSC) populations in the subventricular zone (SVZ) lining the lateral ventricles and in the hippocampus [15,20,21]. These cells express glial acidic fibrillary protein (*gfap*) [22,23] and possess many characteristics shared with astrocytes [22,24]. Perturbations in the Shh signaling pathway impair proliferation and maintenance of these adult NSC populations [17,25,26,27], consistent with its roles in neurodevelopment. Using genetic tools, Shh activity was later identified in discrete populations of astrocytes throughout the brain [13,14]. Indeed, the vast majority of cells transducing Shh signaling in adulthood correspond to mature, differentiated astrocytes. *Shh* is expressed by neurons [13,14,28], identifying Shh signaling as a molecular pathway mediating neuron–astrocyte communication. Indeed, a growing body of evidence suggests it is involved in complex and multifaceted cell–cell interactions that support modulation of synaptic organization and function in the healthy brain.

Transduction of Shh signaling is initiated by Shh binding to its receptor patched 1 (PTC1), a 12-pass transmembrane receptor. Binding of Shh to PTC1 relieves inhibition of a second transmembrane protein, smoothened (SMO), a member of the G protein-coupled receptor (GPCR) family. Three GLI proteins, GLI1, GLI2, and GLI3, belonging to the family of zinc-finger transcription factors, are the primary transcriptional effectors of Shh signaling in vertebrates and possess both activator and repressor functions. Activation of SMO promotes the transport of full-length, activated GLI2 to the nucleus where it subsequently promotes transcription of Shh target genes, including *Gli1*. Because *Gli1* activation occurs only in the presence of high levels of Shh activity, its transcriptional activation serves as a reliable readout of Shh signaling [29]. In the absence of Shh, GLI2 and GLI3 are truncated by post-translational proteolytic processing that represses transcription. Comprehensive reviews on the molecular mechanics of Shh signaling are available, and the reader is referred to some of these here [30,31,32].

Astrocytes encompass a complex and functionally diverse population of cells and are now recognized for their essential roles in a broad array of nervous system functions. Astrocytes are required for synapse formation [33,34] and are increasingly implicated in neural circuit organization and function [35,36,37,38,39]. They also regulate homeostatic levels of ions, such as K^+,^ and are responsible for rapid clearance of neurotransmitters such as glutamate from the synapse [40,41,42]. Together with endothelial cells and pericytes, astrocytes promote blood–brain barrier (BBB) integrity, limiting access of blood-borne molecules and cells to CNS parenchyma [43]. Beyond their roles in the healthy CNS, astrocytes respond to nearly all manner of CNS damage, including acute insults such as stroke or spinal cord injury, as well as chronic neurodegenerative disorders such as Alzheimer’s disease [44]. Astrocytes produce and respond to inflammatory cytokines and facilitate the recruitment or restriction of inflammatory cells to sites of injury. Given the breadth of astrocyte contributions to CNS function, much remains to be discovered regarding the underlying molecular mechanisms responsible for orchestrating such diverse functional properties. There is growing evidence that Shh signaling in astrocytes plays an important role in regulating a number of these functions, from synapse organization to restricting inflammation.

## 2. Shh Signaling in the Adult Brain

**Figure 1:**
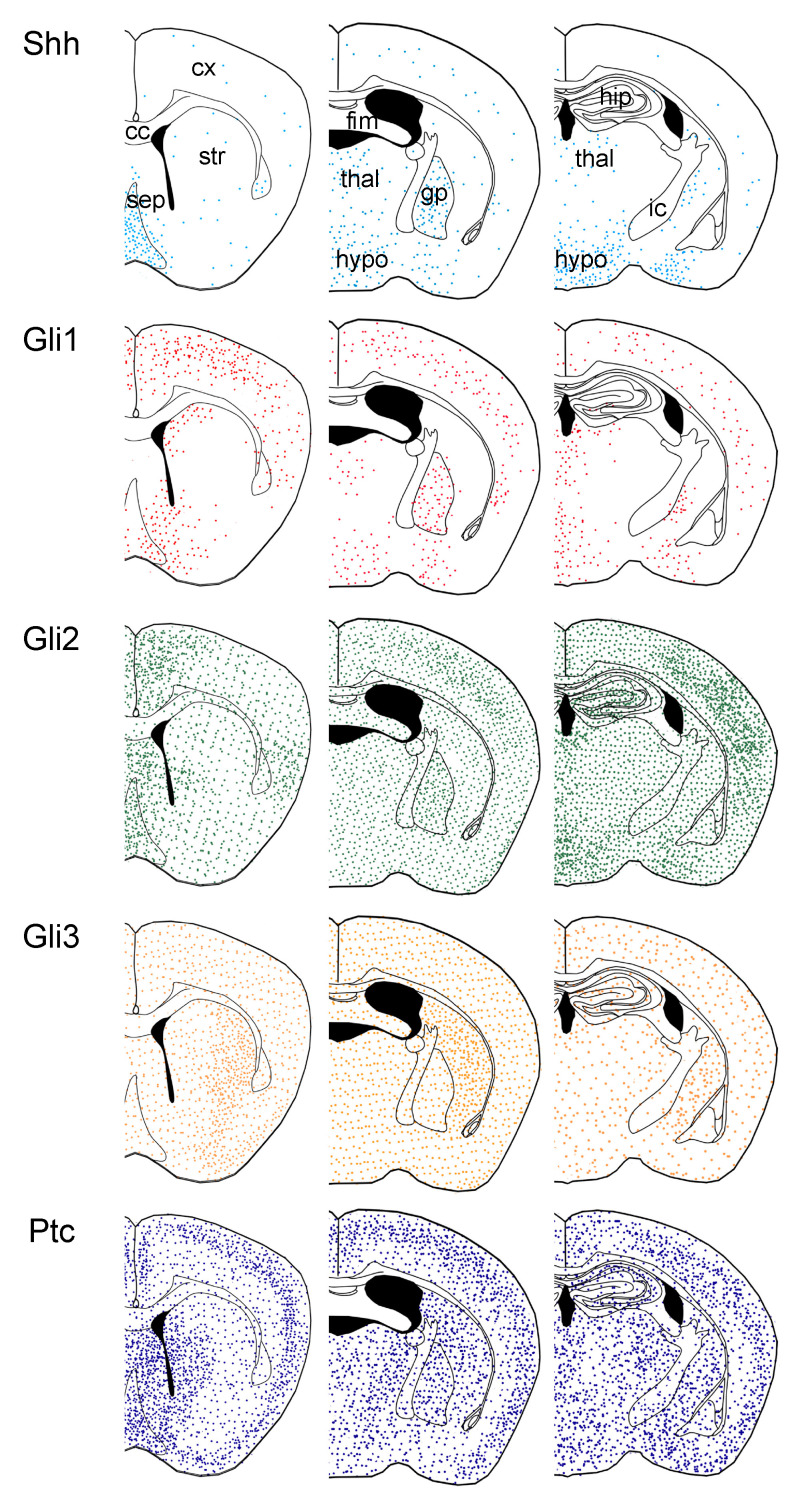
Schematic depicting the relative distribution of various components of the Shh signaling pathway in the intact, adult forebrain across the anterior–posterior axis. Data derived from genetic labeling, as reported by Garcia et al. (2010), and in situ hybridization studies [18,19,45]. Note the relative concordance in distribution of cells expressing *Shh* and *Gli1*, whereas cells expressing *Gli2*, *Gli3*, and *Ptc* are found in several regions where *Gli1* expression is absent. *Shh* is expressed by neurons, whereas *Gli1*, *Gli2*, *Gli3*, and *Ptc* are expressed by astrocytes [14]. cc, corpus callosum; cx, cortex; fim, fimbria; gp, globus pallidus; hip, hippocampus; hypo, hypothalamus; ic, internal capsule; sep, septum; str, striatum; thal, thalamus.

Early work mapped multiple components of the Shh signaling pathway throughout the adult brain by in situ hybridization [18,19], establishing its persistence in the nervous system beyond neurodevelopment. Transcripts for *Shh* were found in ventral forebrain structures, such as the hypothalamus and septum, and most prominently in cerebellar Purkinje cells. *Shh* transcripts were also detected in cells residing in deep layers of the cortex [45]. Transcripts for *Ptc* and *Smo* were also found in these regions, as well as regions that lacked *Shh* transcripts, but were targets of *Shh*-containing regions, suggesting both local and long-distance signaling [46]. Using genetic tools, later studies identified cells expressing *Gli1* in these regions, as well as in adult neural stem cell populations, further confirming transcriptional activity of the Shh pathway throughout the adult forebrain [14,15,27]. Colocalization analysis with cell-type-specific markers established neurons as the source of Shh ligand, whereas *Gli1*-expressing were identified as astrocytes, establishing these cells as the predominant cells transducing Shh signaling outside of the adult neurogenic niches [14] (Figure 1). Consistent with this, astrocytes also express *Gli2*, *Gli3*, and *Ptc* [14], demonstrating that these cells possess the necessary machinery to transduce the pathway. Whereas *Gli1* expression was found predominantly in astrocytes, whether *Gli2*, *Gli3*, and *Ptc* are similarly restricted to astrocytes is not known. Noncanonical, Gli-independent Shh signaling occurs in neurons [28,47], suggesting that Ptc and Smo are likely expressed in these cells. *Gli1*-expressing astrocytes are most abundant in ventral regions, such as the hypothalamus, amygdala, and septum, but are also found in substantial fractions of astrocytes in the globus pallidus and in the neocortex [14,18]. In contrast, *Gli1*-expressing astrocytes are notably absent in the striatum and in white matter tracts [14] (Figure 1). This characteristic distribution may reflect functionally distinct populations of cells. Alternatively, this may instead reflect repression of Shh activity in a regionally defined manner. Indeed, other components of the pathway, including *Gli2*, *Gli3*, and *Ptc* exhibit a much broader distribution in the adult brain than *Gli1* and are found in regions where *Gli1* is noticeably absent, such as in the striatum [14] (Figure 1). Notably, *Gli3*, a transcriptional repressor of Shh activity, is found in the striatum and in white matter, which are largely devoid of cells expressing *Gli1*, suggesting that Shh signaling is actively repressed in these regions. Thus, the distribution of multiple components of the pathway suggests that many more cells possess the machinery to transduce Shh signaling than might be expected based on *Gli1* expression alone. Shh exerts powerful mitogenic functions, and the activity of the pathway must be tightly regulated since aberrant activation is associated with tumor formation [48]. Disentangling the physiological roles of Shh signaling in astrocyte function from its tumorigenic properties would be interesting for future studies to address.

Although Shh signaling is found in many regions throughout the brain, activity of the pathway varies across regions. The proportion of astrocytes expressing *Gli1* differs across regions. In the cortex, ~25–30% of astrocytes express *Gli1* [14,49], whereas the vast majority of astrocytes in the hypothalamus are *Gli1*-positive [14,50]. This is generally correlated with the abundance of *Shh*-expressing neurons in some regions. In the hypothalamus and globus pallidus, for example, the number of neurons expressing *Shh* is relatively high (Figure 1). This heterogeneity in activity of the pathway between regions suggests molecular heterogeneity and differential regulation of astrocytes both between regions and within a given region. Indeed, the functional diversity of astrocytes is of intense interest; however, the molecular identity and regulation of distinct astrocyte populations remain poorly understood [37,51,52,53]. It is interesting to consider whether Shh signaling confers unique functional properties or genetic signatures in specific populations of astrocytes.

Cells expressing *Shh* are most abundant in the hypothalamus and medial septum but are also found in the neocortex. In the cerebellum, Purkinje cells produce Shh that is received by neighboring Bergmann glia [13]. The precise mechanism by which *Shh*-expressing neurons release Shh and communicate to astrocytes is not well understood. There is a close correspondence between *Shh* and *Gli1*, with regions showing a high abundance of *Shh*-expressing cells also showing a high abundance of *Gli1*-expressing cells, such as in the hypothalamus, suggesting that Shh may be released locally, mediating local communication between neurons and neighboring astrocytes. However, Shh may also be transported axonally, indicating the potential for long-range communication between these cells. *Shh* transcripts have been detected in retinal ganglion cell axons [9,54,55]. In addition, although astrocytes in the striatum do not express *Gli1*, conditional deletion of *Shh* from midbrain dopaminergic neurons leads to a progressive degeneration of midbrain TH+ cells as well as their fast-spiking, cholinergic striatal targets [47], suggesting that noncanonical, Gli-independent Shh activity occurs in these neurons.

## 3. Shh Signaling in Astrocyte Modulation of Synapses

The last twenty or so years in neuroscience have seen renewed interest in and heightened attention on the role of astrocytes in synaptic function. Despite their former reputation as simple support cells responsible for providing trophic support for neurons, astrocytes are now recognized as a key cellular element of synapses [33,56]. Astrocyte processes ensheath synapses [57,58] and are enriched with various channels and transporters that promote healthy synaptic function. Clearance of glutamate from the synapse is performed predominantly by astrocytes through the glutamate transporters GLT1 and GLAST1 [59,60], which are found on astrocyte processes. Likewise, astrocytes regulate extracellular K^+^ homeostasis, through Kir4.1, an inwardly rectifying K^+^ channel [61]. Pioneering work near the turn of the century demonstrated the requirement of astrocytes for synapse formation and function *in vitro* [34,62]. Significant advances have since been made identifying a number of astrocyte-derived molecules that are required to establish, maintain, and ensure proper function of synapses [33].

Although the gene expression programs regulated by Shh signaling in astrocytes are not yet well understood, studies using cell-type-specific gene deletion strategies to selectively perturb Shh signaling in astrocytes have identified key synapse-associated genes that are Shh-dependent. Most notably, Kir4.1, encoded by the gene KCNJ10, is dysregulated in mice with astrocyte-specific deletions of *Smo* [13,49]. Kir4.1 facilitates the rapid clearance of K^+^ ions from the synapse, ensuring appropriate extracellular [K^+^] necessary for appropriate neuronal firing. Loss of Kir4.1 increases neuronal excitability [63,64]. Selective deletion of *Smo* in astrocytes leads to a reduction in Kir4.1 expression accompanied by heightened excitability of neurons [13,49]. In addition, Shh signaling is required for expression of the AMPA receptor subunits GluA1 and GluA4 as well as the glutamate transporter GLAST in Bergmann glial cells of the cerebellum [13]. Ectopic Shh activity in cerebellar velate astrocytes, which normally exhibit low levels of Shh activity, causes them to adopt a transcriptional profile that more closely resembles that of Bergmann glia, including increased expression of GluA1 and GluA4 [13], suggesting that Shh signaling is sufficient to promote expression of genes associated with synapse function.

Astrocyte modulation of synaptic plasticity and organization also requires Shh signaling. In the neocortex, selective deletion of *Smo* in astrocytes leads to an overabundance of dendritic spines selectively in the apical dendrites of deep-layer neurons [49], coinciding with the enrichment of astrocytes expressing *Gli1* in layers 4 and 5. Notably, neurons in the upper layers, where Shh activity is largely absent, show no such perturbation in spine density. Chronic, in vivo imaging by two-photon microscopy shows that spines in *Smo* conditional knockout (CKO) mice exhibit increased survival, suggesting that synaptic plasticity is impaired following the loss of Shh activity in astrocytes. Interestingly, these spine phenotypes emerge during postnatal development and persist into adulthood. Aberrant circuit formation and synaptic plasticity characterize a number of neurodevelopment disorders, including fragile X syndrome, Rett syndrome, and autism spectrum disorders (ASDs). The identification of a role for Shh signaling in astrocyte modulation of synaptic organization raises the question of whether targeting Shh activity in astrocytes may provide benefits that mitigate or reverse such aberrant synaptic organization and function.

Astrocyte transduction of Shh signaling is most pronounced in the hypothalamus, in which at least 80% of astrocytes express *Gli1,* coinciding with a high abundance of *Shh*-expressing neurons in this region [14,50]. The hypothalamus plays a central role in key homeostatic behaviors, including appetite and energy expenditure. Genetic activation of Shh signaling by astrocyte-specific ablation of *Ptc* produces lean mice that fail to gain weight with age [50]. Moreover, when challenged with a high-fat diet, these mice fail to show diet-induced weight gain and protection against diet-induced elevated glucose and insulin resistance. Activation of Shh signaling in astrocytes increased expression of insulin receptor selectively in the hypothalamus. These observations demonstrate that Shh signaling regulates energy metabolism and protects against dysregulation of energy metabolism associated with aging and obesity. This is further supported by the observation that *Shh* transcripts in the hypothalamus are reduced in leptin-deficient mice (*Lep^ob^*) [65].

## 4. Astrocyte-Mediated Inflammation and the Role of Shh Signaling

Beyond their roles as modulators of synapse formation and function in the healthy brain, astrocytes have gained increasing recognition as key cellular effectors of both pro- and anti-inflammatory functions. Astrocyte end-feet line blood vessels and produce both pro- and anti-inflammatory signals that actively recruit or limit leukocyte invasion [66]. Functional interactions between endothelial cells and astrocytes form the BBB, restricting the entry of blood-borne leukocytes into CNS parenchyma [43]. Perturbations of BBB integrity, such as those occurring following injury or in certain neurological disorders such as multiple sclerosis, permit the entry of inflammatory molecules and peripheral immune cells that interfere with CNS function or can exacerbate pathological conditions. Growing evidence shows that Shh signaling limits inflammation and promotes BBB integrity.

Ischemic-induced activation of microglia is reduced in mice treated with cyclopamine, a powerful antagonist of SMO [67]. Conversely, activation of Shh activity following middle cerebral artery occlusion reduces levels of *TNFα*, *IL-6*, and *IL-1β* transcripts and increases expression of tight junction proteins ZO-1 and occludin, with a concomitant reduction in permeability of the BBB [68,69]. Following a stab wound, peripheral leukocytes invade parenchymal tissues surrounding the lesion. Application of SAG, an agonist of the Shh pathway, reduces macrophage accumulation in the parenchyma [70]. However, this effect is abolished in mice carrying a selective deletion of *Smo* in astrocytes, suggesting that astrocytes are key cellular effectors of the anti-inflammatory effects of Shh signaling. In support of this, overactivation of Shh signaling specifically in astrocytes, achieved by deleting *Ptc*, lowers *TNFα* levels in the cortex [50]. Pharmacological blockade of Shh activity by the SMO antagonist cyclopamine increases the extravasation of plasma proteins into the CNS *in vivo* [71]. Interestingly, selective deletion of *Smo* in endothelial cells reduces the expression of tight junction proteins such as occludin, claudin-3, claudin-5, and ZO1, with a concomitant reduction in BBB permeability, demonstrating a role for Shh activity in endothelial cell modulation of BBB integrity. The authors further demonstrate a reduction in astrocyte association with blood vessels, suggesting that endothelial cells produce a cue that attracts astrocyte end-feet and that this cue is *Smo*-dependent. This suggests that Shh signaling mediates bidirectional communication between astrocyte end-feet and endothelial cells that promotes BBB structure and function. Identifying the Smo-dependent cues in endothelial cells that regulate astrocyte association with blood vessels will shed light on molecular signals that establish and maintain the BBB, which is essential for restricting inflammation. One candidate is Desert hedgehog (DHH), a member of the hedgehog family of signaling molecules. DHH mediates cell–cell communication between endothelial cells and promotes BBB function [72]. Endothelial cell-specific deletion of DHH increases BBB permeability at the endothelial cell barrier while decreasing astrocyte permeability, restricting inflammatory cells that escape the endothelial barrier to the perivascular space. These observations demonstrate that Hh signaling mediates complex interactions between endothelial cells and astrocytes that promote BBB integrity, while also highlighting the need for further studies to dissect the precise roles of the pathway in different cell types.

Interestingly, several studies have identified astrocytes as the source of Shh in the brain following injury [67,71,73,74]. However, genetic labeling studies in *Shh^CreER^* mice identified *Shh* only in neurons and did not detect transcriptional activation of *Shh* in any astrocytes [13,14]. In support of this, a recent study using single-cell RNA sequencing of cells associated with brain vasculature showed an absence of *Shh* transcripts in astrocytes [75]. One possibility is that the injured environment stimulates *Shh* expression in astrocytes. Injury studies using genetic labeling and fate mapping approaches are needed to better understand the cellular source of Shh under pathological conditions. Nevertheless, it is clear that Shh signaling limits inflammation through its actions in astrocytes and endothelial cells. In addition, inflammation itself may also regulate Shh activity. IL-1β reduced *Shh* mRNA in cultured astrocytes [76]. *In vivo*, transcriptional activation of *Gli1* is reduced in astrocytes as early as 24 hours following injury, but it is restored to baseline levels two weeks later [70], as injury-induced inflammation resolves [77]. These studies identify a role for Shh signaling in astrocyte-mediated regulation of inflammation and suggest this pathway may serve as a potential target for therapeutic interventions against inflammation in injury or disease. Elucidating the molecular cues that regulate the activity of the pathway would advance our understanding of the precise mechanism by which Shh signaling exerts its anti-inflammatory benefits in the nervous system.

## 5. Shh Signaling in Endogenous Progenitors and Reactive Gliosis after Injury

The mitogenic properties of Shh signaling in the developing and postnatal CNS are well established. Shh activity promotes proliferation of neural precursors in the embryonic spinal cord and granule cell precursor cells in the cerebellum [12,78,79]. In the adult CNS, proliferation and maintenance of neural stem cells (NSCs) residing in the subventricular zone (SVZ) and hippocampus are similarly Shh-dependent [20,21,25,27]. Outside these neurogenic niches, however, most neural cells, including astrocytes, are postmitotic in the healthy and intact CNS. However, injury can stimulate proliferation of both endogenous progenitors residing within the neurogenic niches of the adult brain and local cells at or near the injury site, and growing evidence shows a role for Shh signaling in regulating injury-induced proliferation.

Consistent with its mitogenic properties in the developing CNS, early studies demonstrated that application of Shh or agonists of the pathway increase proliferation of local endogenous progenitor cells following contusion or demyelinating injuries of the spinal cord [80,81]. Similar observations have now been reported following various injury models, including ischemia, traumatic brain injury, or kainic acid injection [67,68,82]. Conversely, pharmacological agents that block pathway activity reduce proliferation [67,83,84]. Cell type analysis of proliferating cells shows that Shh signaling stimulates proliferation of local oligodendrocyte progenitors [73,85,86] and promotes proliferation of adult NSCs in the SVZ and hippocampus [84,87]. Importantly, in studies of demyelinating lesions, there is evidence that Shh signaling in adult NSCs promotes structural and functional neural repair. Fate mapping experiments following a demyelinating lesion in the corpus callosum show that *Gli1*-expressing NSCs are recruited into the lesion and differentiate into myelinating oligodendrocytes and that Shh activity promotes their proliferation [86,88,89]. Interestingly, SVZ-derived Gli1-expressing NSCs downregulate *Gli1* expression upon arrival in the corpus callosum, and genetic ablation of *Gli1* improved remyelination, suggesting that transcriptional silencing of *Gli1* activity promotes remyelination [88]. Pharmacological stimulation of *Gli1* activity with the agonist GANT61 in experimental autoimmune encephalitis (EAE) mice enhanced myelination, improved neuronal survival, and lowered the clinical score of mice during relapse.

In addition to neural stem and progenitor cells, astrocytes can also exhibit injury-induced proliferation, together with other complex behaviors in response to injury. These behaviors are collectively referred to as reactive astrogliosis and include dramatic changes in morphology, gene expression, production, and response to various molecular cues [44]. Reactive astrocytes exhibit context-dependent behaviors that vary with respect to lesion type, severity, and distance from focal insults. In severe injuries, such as ischemia or spinal cord injury, reactive astrocytes at the injury site proliferate, producing glial scar borders that effectively isolate blood-borne immune cells and restrict their invasion into CNS parenchyma [90]. The well-established effects of Shh signaling on the proliferation of various neural progenitor populations suggest that the proliferation of reactive astrocytes is similarly regulated. In support of this idea, upregulation of Shh activity has been observed in various injury models, including spinal cord injury, ischemia, traumatic brain injury, and demyelination [67,73,84,91,92,93]. Interestingly, noninvasive injuries, such as neurodegeneration, fail to increase *Shh* expression and do not trigger the proliferation of reactive astrocytes [94]. Reactive astrocytes isolated from the cortex following a stab wound generate neurospheres *in vitro* [94]. The addition of Shh to the cultures increases the number of neurospheres generated, whereas administration of cyclopamine, a selective antagonist of Shh signaling, to mice abrogates neurosphere formation. These observations demonstrate the requirement for Shh activity in reactive astrocyte proliferation *in vitro* and further suggest that Shh signaling may confer NSC-like properties on these cells.

Unexpectedly, however, mice carrying a genetic deletion of *Smo* selectively in *Gfap*-expressing cells (Gfap Smo CKO) show no difference in proliferation following a cortical stab wound [70]. Further, genetic labeling of cells expressing *Gli1* shows a pronounced reduction in the number of *Gli1*-expressing cells following injury. Because high levels of Shh activity stimulate transcriptional activation of *Gli1*, the application of these genetic tools provides a reliable readout of Shh activity in the injured microenvironment. Transgenic reporter mice carrying the *lacZ* gene targeted to the Gli1 locus (*Gli1^lacZ/+^*) show that as early as 24 hours after stab wound, there are fewer *Gli1*-expressing cells in the cortex [70], demonstrating downregulation of Shh activity after injury. These observations are supported by studies in which *Gli1*-expressing cells are labeled by tamoxifen administration after injury. Using this approach, fewer *Gli1*-expressing cells are labeled by tamoxifen after both traumatic brain injury and cuprizone-induced demyelination when compared to tamoxifen administered to control, uninjured mice [70,87,95]. These observations argue against a role for Shh signaling in the proliferation of reactive astrocytes following injury and suggest that Shh activity is repressed, at least during the acute phases of injury. This contrasts with the studies discussed above and highlights the need for further studies to understand the nature of the pathway in pathological circumstances.

## 6. Outstanding Questions

Although numerous independent studies now demonstrate a role for Shh signaling after injury, conflicting reports in the literature leave open several questions surrounding the precise nature of Shh activity in the injured or diseased CNS. Most notably, whether Shh signaling is upregulated or downregulated after injury and the cellular source of Shh after injury remain poorly understood. In general, studies reporting an increase in Shh activity and localization of the ligand in astrocytes have relied primarily on antibody staining for Shh [68,71,73,91], whereas studies reporting a reduction of Shh activity and localization of ligand in neurons have relied on genetic tools [13,70,87]. Because Shh is secreted, the possibility exists that antibody recognition of Shh on astrocytes may result from recognition of Shh bound to the surface of astrocytes. Notably, antibodies to Shh have been shown to recognize other members of the hedgehog signaling family, Indian hedgehog (IHH) and Desert hedgehog (DHH) [96]. Although *Shh* is the predominant member of the Hh family in the CNS, expression of *Dhh* was reported in CNS endothelial cells in vitro and in human and mouse tissues [72]. Alternatively, these conflicting results may reflect discordance between protein and transcript. The development of novel tools, including specific antibodies that reliably and specifically detect Shh and its molecular components, such as PTC, SMO, and the GLI proteins, and that are validated against genetic models, together with the application of genetic models or high-resolution RNA sequencing in various injury models would advance our understanding of Shh signaling in astrocyte-mediated inflammation. Such work would facilitate the development of novel therapeutic strategies aimed at mitigating the neurological deficits associated with neuroinflammation.

The studies discussed here outline diverse roles for Shh signaling in astrocytes in health and in pathological circumstances. Most injury studies have focused on the inflammatory or proliferative response of astrocytes. However, it will be interesting to examine whether Shh signaling plays a role in astrocyte modulation of synaptic reorganization following injury.
